# Supplementary material for: Read mapping on de Bruijn graphs
Source: BMC Bioinformatics. 2016 Jun 16;17:237. doi: 10.1186/s12859-016-1103-9 (PMC4910249; doi:10.1186/s12859-016-1103-9)
Supplement: Additional file 1 — Read mapping on De Bruijn graphs additional file. Three complementary tables are presented. Main characteristics of data sets used in this study. Assembly and mapping approach comparison. Results of BGREAT on real read sets. (PDF 40 kb) [file 12859_2016_1103_MOESM1_ESM.pdf]

Read mapping on De Bruijn graphs
Additional file

Limasset et. al.

May 24, 2016

Id	Species	nb reads	mean read length	coverage
SRR959239	*E.Coli*	5,372,832	98	114X
SRR065390	*C.Elegans*	67,617,092	100	68X
SRR1522085	*C.Elegans*	46,299,804	51	12X
SRR345593 and SRR345594	*Human*	3,040,306,840	100	95X

Table 1: Main characteristics of data sets used in this study. Presented read numbers are not the same than those in input of *GGMAP* because we only try to align read without 'N'

				Mapping on assembly	Mapping on CDBG
Reads set	Assembler	k	c	Mapping rate	Mapping rate
E.coli	Velvet	31	3	95.57%	97.17%
//	Minia	//	//	96.19%	//
C.elegans	Velvet	21	2	56.33%	89.15%
//	Minia	//	//	51.43%	//
//	Velvet	31	3	80.60%	92.34%
//	Minia	//	//	80.43%	//
Human	Minia	31	10	63.16%	85.51%

Table 2: Assembly and mapping approach comparison . Columns k and c are the assembly main parameters for Velvet and Minia (k is the length of k-mers and c the minimum coverage for a k-mers to be considered). The last column recalls the overall rate of reads mapped using *GGMAP* on the CDBG obtained with the same read sets and k and c parameters. Because of limited computational resources, Velvet was not tested on the Human data-set. The '//' symbol indicates same value(s) between two consecutive lines.

Graph	Mapped set (nb reads)	Mapping Strategy	Nb reads mapped (on branching parts of DBG)	Nb reads fully mapped on unitigs	Overall nb mapped reads
E.coli	SRR959239 (5,128,790)	Greedy	687,074 (13.40%)	4,296,710 (83.78%)	4,983,784 (97.17%)
"	"	Complete	688,929 (13.43%)	4,295,814 (83.76%)	4,984,743 (97.19%)
*C.elegans*_cpx	SRR065390 (67,617,092)	Greedy	44,686,355 (66.09%)	15,592,918 (23.06%)	60,279,273 (89.15%)
*C.elegans*_norm	"	Greedy	13,994,715 (20.70%)	48,442,146 (71.64%)	62,436,861 (92.34%)
*C.elegans*_norm	SRR1522085 (22,509,110)	Greedy	3,523,416 (15.65%)	16,682,194 (74.11%)	20,205,610 (89.77)%
Human	SRR345593 and SRR345594 (2,967,536,821)	Greedy	1,004,182,363 (33.84%)	1,533,456,046 (51.67%)	2,537,638,409 (85.51%)

Table 3: Results of *BGREAT* on real read sets. We remind that the column "*Nb reads fully mapped on unitigs*" corresponds to the number of reads mapped by Bowtie2 with defaults parameters. We also remind that reads from SRR1522085 are mapped on the CDBG obtained using reads from read set SRR065390. For all other lines, the same read set was used both for constructing the CDBG and during the mapping.
